# Obituary

**Published:** 1888-02

**Authors:** 


### Obituary.

Died suddenly at his home in Madisonville, Ky., December 31st, 1887, Dr. James H. Prewitt, D.D.S.

Dr. Prewitt was one of the prominent members of the Dental Profession in Kentucky. He was about forty years old; was the son, and probably the grandson of a dentist. The young manhood of his life he spent with his father at Paducah, Ky. About 1865 he removed to his present office at Madisonville. He some years ago received the D.D.S. (honorary) from the Ohio College of Dental Surgery. He was a successful practitioner, being trained in his father's office, he was familiar with all that pertained to it. Although his home was in Kentucky he was in one sense at least about as much at home in Tennessee, for he was as well known in the latter as in the former, and was held in equally as high esteem by the profession there. He was a man of more than ordinary ability and attainments. He was much interested in matters outside of his profession. He was a good historian, and took great interest in political affairs. He was in several respects a representative man; and one of whom much was expected in the future.

According to human views such men are greatly needed in the world, and can ill be spared, but it becomes us to bow to the will and ways of Him who holds all destiny in His hands, and disposes of all events as He will.

